# Optimizing Patient-Reported Outcome Collection and Documentation in Medical Music Therapy: Process-Improvement Study

**DOI:** 10.2196/46528

**Published:** 2023-07-27

**Authors:** Samuel N Rodgers-Melnick, Seneca Block, Rachael L Rivard, Jeffery A Dusek

**Affiliations:** 1 University Hospitals Connor Whole Health Cleveland, OH United States; 2 Department of Population and Quantitative Health Sciences School of Medicine, Case Western Reserve University Cleveland, OH United States; 3 Department of Psychiatry School of Medicine, Case Western Reserve University Cleveland, OH United States; 4 Center for Survey and Evaluation Research HealthPartners Institute Minneapolis, MN United States; 5 Department of Family Medicine and Community Health School of Medicine, Case Western Reserve University Cleveland, OH United States

**Keywords:** electronic health record, integrative medicine, music therapy, pain, quality improvement

## Abstract

**Background:**

To measure the effectiveness of nonpharmacologic interventions delivered during clinical care, investigators need to ensure robust and routine data collection without disrupting individualized patient care or adding unnecessary documentation burden.

**Objective:**

A process-improvement study was undertaken to improve documentation consistency and increase the capture of patient-reported outcomes (PROs; ie, stress, pain, anxiety, and coping) within a medical music therapy (MT) team.

**Methods:**

We used 2 Plan-Do-Study-Act (PDSA) cycles to improve documentation processes among an MT team (13.3 clinical full-time equivalent staff). Trainings focused on providing skills and resources for optimizing pre- and postsession PRO collection, specific guidelines for entering session data in the electronic health record, and opportunities for the team to provide feedback. Two comparisons of therapists’ PRO collection rates were conducted: (1) between the 6 months before PDSA Cycle 1 (T0) and PDSA Cycle 1 (T1), and (2) between T1 and PDSA Cycle 2 (T2).

**Results:**

Music therapists’ rates of capturing any PRO within MT sessions increased significantly (*P*<.001) from T0 to T1 and from T1 to T2 for all domains, including stress (4/2758, 0.1% at T0; 1012/2786, 36.3% at T1; and 393/775, 50.7% at T2), pain (820/2758, 29.7% at T0; 1444/2786, 51.8% at T1; and 476/775, 61.4% at T2), anxiety (499/2758, 18.1% at T0; 950/2786, 34.1% at T1; and 400/775, 51.6% at T2), and coping (0/2758, 0% at T0; 571/2786, 20.5% at T1; and 319/775, 41.2% at T2). Music therapists’ feedback and findings from a retrospective analysis were used to create an improved electronic health record documentation template.

**Conclusions:**

Rates of PRO data collection improved within the medical MT team. Although the process improvement in this study was applied to a nonpharmacologic MT intervention, the principles are applicable to numerous inpatient clinical providers. As hospitals continue to implement nonpharmacologic therapies in response to the Joint Commission’s recommendations, routine PRO collection will provide future researchers with the ability to evaluate the impact of these therapies on pain relief and opioid use.

## Introduction

To provide evidence-based, patient-centered care to hospitalized patients, health care professionals should evaluate the impact of their interventions on patient-reported outcomes (PROs) such as stress, pain, anxiety, and patients’ ability to cope with the stressors of hospitalization. PROs are vitally important at every level of health care delivery, from understanding changes within individual patients to communicating the impact of interventions to health care teams, administrators, payors, and the global research community [1].

These PROs have become increasingly important in the wake of the opioid epidemic as hospitals shift their pain management approaches from relying on opioid medication toward promoting and providing evidence-based nonpharmacologic pain treatment in accordance with Joint Commission guidelines [2-5]. In the context of inpatient integrative therapies provided for pain relief (eg, acupuncture, massage, and music therapy [MT]), PROs are an important measure of value within the health care system. PROs demonstrate whether patients’ symptoms, quality of life, and physical function are improving in response to treatment [6,7]; facilitate shared care and decision-making with the medical team [8]; and can improve patient empowerment and overall satisfaction with health care [8]. Use of PROs within interventions allows health care professionals to identify the need for modifications in the treatment plan (eg, using an active music-making MT intervention instead of a receptive MT intervention) and determine whether further action is needed to improve patients’ self-efficacy for managing their conditions [1]. Routine PRO collection is also essential within practice-based research for evaluating the effectiveness of nonpharmacologic therapies across health care systems [6,9,10].

Despite the importance of PROs in patient care and research, implementing routine PRO collection among health care professionals has been limited by factors including clinician, staff, and patient reluctance; concerns for how the data will be used; and technology challenges related to the workflow within the electronic health record (EHR) [7]. To measure the effectiveness of nonpharmacologic therapies, such as MT, investigators need to ensure robust data collection without disrupting individualized patient care or adding unnecessary documentation burden for therapists. Previous studies have described processes for improving PRO collection within nursing [11], oncology [12], outpatient integrative health and medicine [6], and a pediatric psychology service for children with sickle cell disease [13]. However, to our knowledge, no studies have described or evaluated processes for improving PRO collection among a medical MT team.

We are currently conducting a large research project entitled *Effectiveness of Medical Music therapy Practice: Integrative Research using the Electronic health record* (EMMPIRE). EMMPIRE is an observational study with three aims: (1) a retrospective study examining single-session clinical effectiveness in hematology and oncology within an academic cancer center [14] and 8 community hospitals [15]; (2) a quality improvement initiative to improve documentation consistency and increase the routine collection of PROs; and (3) a prospective study to further understand the clinical effectiveness of MT on health care use (eg, length of stay and pain medication use) and longitudinal changes in PROs.

During the EMMPIRE Aim 1 retrospective study of over 15,000 MT sessions, the investigators identified several needs for improvement within MT documentation, including (1) adding new PROs (eg, stress and coping) to measure domains for which MT was indicated; (2) increasing rates of routine PRO collection; and (3) providing structured data entry for free-text fields related to MT session characteristics. Therefore, a process-improvement study was conducted to determine if it was possible to improve documentation consistency and increase the routine collection of PROs within a medical MT team.

## Methods

### Design and Participants

**Figure 1:**
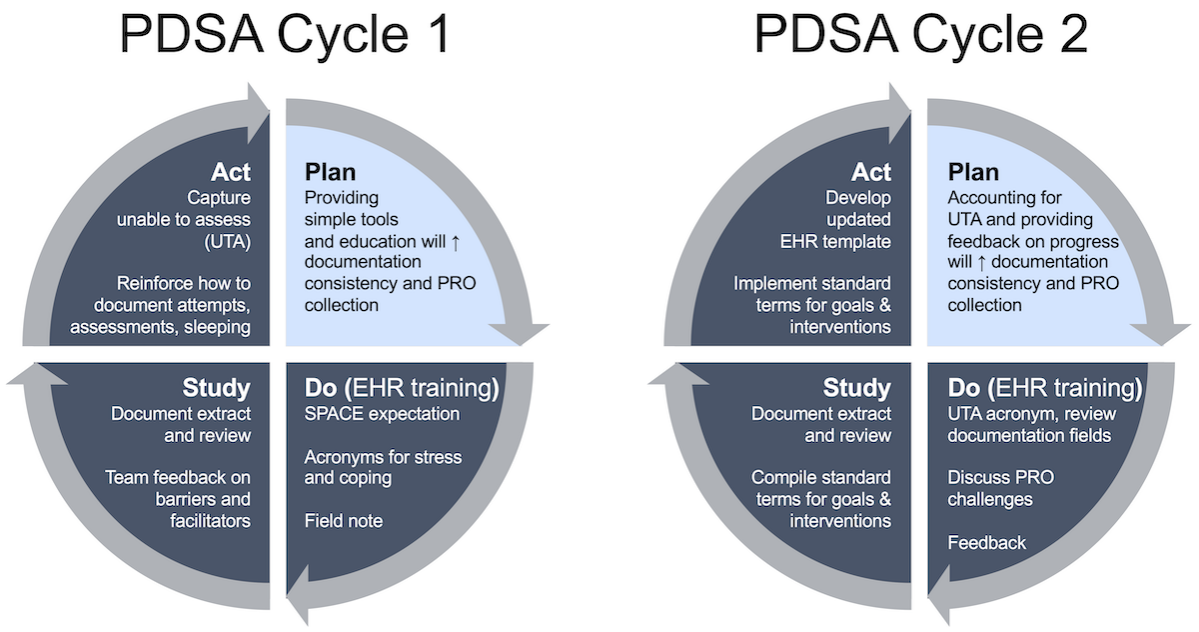
Plan-Do-Study-Act (PDSA) Cycles. Two PDSA cycles were implemented between July and December 2020 to improve assessment, evaluation, and documentation processes among a music therapy team. EHR: electronic health record; PRO: patient-reported outcomes; SPACE: stress, pain, anxiety, coping, education; UTA: unable to be assessed.

We implemented 2 Plan-Do-Study-Act (PDSA) cycles [16] between July and December 2020 to improve assessment, evaluation, and documentation processes among an MT team. PDSA cycles are valuable quality improvement tools designed to (1) establish a plan for change, (2) execute that plan, (3) evaluate the outcome of the intervention, and (4) develop a final plan through the synthesis of the information generated during the process [17]. Our PDSA cycles (see Figure 1) addressed common barriers to routine PRO collection and documentation during MT sessions, with the primary goal to continually increase the proportion of PRO collection over 6 months. At the time of the process-improvement study, the MT team included 10.3 full-time equivalent (FTE) board-certified music therapists and 3 FTE MT interns (13.3 total FTE). Periodic retrospective EHR reviews were conducted to monitor therapists’ rates of PRO collection.

### Setting

University Hospitals (UH) is a not-for-profit health system in Northeast Ohio serving the needs of more than 1.2 million unique patients annually. UH Connor Whole Health (UHCWH), a center for integrative health and medicine embedded within the UH health system, partners with UH physicians, providers, and institutes to meet the growing demand for the comprehensive treatment of chronic health conditions and overall well-being. UHCWH seeks to weave integrative health and medicine modalities throughout the fabric of the entire health system. Accordingly, UHCWH includes an expressive therapies program consisting of board-certified music therapists and art therapists**.** At the time of this study, the UHCWH Expressive Therapies Program provided MT (over 10,000 sessions per year) across 10 of UH’s 18 medical centers, including an academic medical center, a freestanding cancer center, and 8 community hospitals.

Within each of the medical centers, music therapists routinely collaborate with the medical care team (eg, physicians, advanced practice providers, nurses, social workers, and chaplains) to address patients’ symptoms and enhance psychosocial support. This program has been integrated within the clinical care team infrastructure as a nonpharmacologic resource for symptom management. Additionally, this inpatient MT program has been used to offer education (eg, verbal and written descriptions of services) on available outpatient UHCWH integrative health and medicine modalities, including chiropractic care, massage therapy, acupuncture, and integrative medicine consults.

### Ethical Consideration

The EHR review procedures used in this study were approved by the UH Cleveland Medical Center institutional review board as part of a retrospective chart review with a waiver of informed consent (STUDY20191213). This study was conducted in accordance with the World Medical Association Declaration of Helsinki.

### PRO Measures

The MT team was instructed to collect 0-10–point numeric rating scale (NRS) measures of stress, pain, anxiety, and coping before (presession) and after (postsession) providing an MT session. The NRS is a validated measure for acute pain intensity [18]. It has been widely used within studies of integrative therapies [10] and found to be more reliable than the visual analog scale in clinical trials, especially among patients of low socioeconomic status [10]. Investigators in previous studies have also used the 0-10 NRS to measure other domains, including anxiety in clinical effectiveness studies of nonpharmacologic interventions (eg, acupuncture, massage therapy, and meditation) [19-21] and stress in a randomized controlled trial of MT [22]. For the NRS of pain, stress, and anxiety, patients were asked, “How much (stress, pain, or anxiety) are you having right now?” with 0 signifying “none” and 10 signifying “worst possible.”

Our retrospective study revealed that coping was a common reason for MT referral [23] and a prevalent goal within MT sessions [15]. Thus, it was important to measure coping pre- and postsession to evaluate the effectiveness of MT for addressing patients’ perceived ability to cope with hospitalization. Given the challenges of implementing long, multi-item questionnaires within inpatient care [24], an NRS was chosen to conduct brief, momentary assessments of patients’ perceived coping abilities. In previous studies, investigators have used the NRS to measure changes in coping among women receiving acupuncture following mastectomy [25] and teachers [26-28]. Among teachers, the coping NRS demonstrated sensitivity to detect intervention effects [26]. In our study, patients were asked, “How well are you coping right now?” with 0 signifying “not coping well” at all and 10 representing “coping very well.”

### Role of Research Team

The research team leading this quality improvement initiative included a music therapist and researcher within the MT team (SRM), the manager of the Expressive Therapies Program (SB), a statistician within the UHCWH research team (RLR), and the principal investigator and director of research for UHCWH (JAD).

### PDSA Cycle 1

#### Plan

Before this study, PRO assessment and evaluation were not established as a clinical expectation in all MT sessions. During our retrospective review of MT documentation, it was evident that several MT sessions addressing stress, pain, anxiety, and coping lacked the collection of these PROs. Furthermore, structured data entry fields within the MT EHR documentation template were used inconsistently, making it challenging to aggregate and subsequently analyze data from MT sessions (eg, format, goals, interventions, and outcomes). By providing simple tools, education, and a managerial expectation for collection of PROs, it was posited that documentation consistency and PRO collection would improve within the MT team.

#### Do

Four web-based group trainings were conducted between July and November 2020. The first training focused on setting an expectation for *SPACE*: collecting measures of *stress, pain, anxiety, and coping* and providing *education* about the role of MT services in the hospital. Specifically, music therapists were expected to collect either (1) pre- and postsession PROs for all MT interventions (eg, active music making, music-assisted relaxation and imagery) in which there were no patient limitations (eg, cognitive or physical limitations); or (2) presession PROs only for MT sessions in which the therapist assessed the patient and provided education but did not conduct an MT intervention. The manager of the expressive therapies program (SB) educated the MT team on techniques to approach patients and administer PRO measures verbally during a mandatory staff meeting. This education included a discussion of the importance of PROs for (1) understanding the impact of MT on individual patients; (2) communicating the impact of MT to hospital leadership; (3) investigating the real-world clinical effectiveness of MT throughout the hospital system; and (4) contributing to the evidence base for medical MT.

Additionally, the *Expressive Therapy Healing SPACE Assessment* was provided as a paper field note for therapists to use within sessions to note patient responses in real time. This field note contained (1) defined spaces for therapists to collect PRO data; (2) specific language for assessing PROs; and (3) the specific acronym expansion codes to use when documenting in the EHR. This field note was formatted to match the layout of the MT EHR documentation template so that therapists could easily transfer information from the paper field note to the EHR.

At the time of the study, radio button fields for pain and anxiety NRS were built within the MT EHR documentation template. Since changes to EHR documentation take several months, strategies were implemented to improve MT documentation using acronym expansion codes and free-text paragraph fields within the MT EHR documentation template. The acronym expansion codes (ie, expresspre, StressPre ., CopingPre ., expresspost, StressPost ., and CopingPost .) created defined spaces in the narrative for therapists to enter pre- and postsession PROs. These PROs could then be mined by departmental data analysts using regular expression functions within commercial statistical packages. A step-by-step screenshot example of all procedures was included in an EHR documentation guide that was used to organize virtual team trainings. The EHR documentation guide was continually updated using therapists’ feedback and made available as a web-based resource for the MT team.

#### Study

A retrospective EHR review was undertaken to determine if the proportion of PRO collection had improved. Using clinical performance management tools within the EHR, all MT documents written during the retrospective study period and the first series of trainings were extracted. The extract provided pain and anxiety PROs within specific reportable fields. Then, regular expression functions in RStudio (version 1.3.1073) [29] were used to extract the stress and coping PROs from free-text paragraph fields. The analysis demonstrated that although the overall PRO collection rates had improved, rates of collecting stress, anxiety, and coping PROs were still less than 50% among all documented MT sessions. In addition to the PROs, there were inconsistencies in documenting (1) conflicts of service (ie, an attempt was made to see a patient but a session did not occur due to the patient being away from their room, asleep, busy, etc); (2) sessions in which the music therapist assessed the patient and provided education but did not conduct an MT intervention; and (3) sessions in which the patient fell asleep in response to an MT intervention.

In addition, 2 feedback sessions were conducted with the MT team to discuss barriers and facilitators to PRO collection and EHR documentation. Facilitators included (1) use of the field note, which provided a concrete reminder to collect PROs and a formatting structure that facilitated seamless data entry in the EHR; (2) having a laminated form patients could use to circle their PROs; and (3) discussing postsession PROs with patients to allow them to recognize their responses to MT. The MT team identified barriers to routine PRO collection among patients who are frustrated, withdrawn, or tangential in conversation. The MT team noted, importantly, that it was not possible to collect PROs among patients experiencing cognitive impairment, emotional distress, or certain physical limitations (eg, tracheostomy or sedation). The MT team expressed a desire to account for those sessions in which PROs are unable to be assessed (UTA) due to these patient limitations. Some MT team members also discussed challenges incorporating NRS measures within their therapeutic style and routine verbal skills for assessment and rapport building within MT sessions.

#### Act

After reviewing the MT documentation in PDSA Cycle 1 and receiving feedback from the MT team, a plan was established to capture instances in which PROs were UTA and reinforce training on documentation procedures. Additional trainings were also planned to provide instruction on how to incorporate PRO collection within routine verbal skills for assessment and rapport building, especially among patients who are frustrated, withdrawn, or tangential in conversation.

### PDSA Cycle 2

#### Plan

Based on the knowledge gained from PDSA Cycle 1, it was posited that further improvements in documentation consistency and PRO collection were possible through (1) additional training on verbal skills for PRO collection and EHR documentation; (2) accounting for instances of outcomes UTA in rates of PRO collection; and (3) providing feedback to the MT team on how PRO collection can contribute to greater understanding and appreciation of MT’s clinical effectiveness for reducing symptom burden and improving coping.

#### Do

Four additional web-based group trainings were delivered between November and December 2020. The first training reinforced specific guidance for EHR documentation including how to document sessions in which (1) there was a conflict of service; (2) only assessment and education were provided; or (3) patients fell asleep in response to MT. Like the methods for documenting stress and coping, an acronym expansion code was created for documenting one of six reasons for outcomes UTA: (1) not applicable (the outcome was not applicable within the MT session); (2) cognitive limitation (the patient’s cognitive limitations such as dementia, confusion, or agitation prevented the patient from providing NRS); (3) physical limitation (the patient’s physical limitations such as tracheostomy, sedation, or aphasia prevented the patient from providing NRS); (4) declined (the patient declined to rate NRS); (5) emotional distress (the patient was in too much emotional distress to provide NRS); and (6) other (the MT was unable to collect NRS for some other reason such as the session being interrupted). The updated field note with UTA codes was provided to therapists to reinforce these changes.

In subsequent trainings, feedback was provided to the MT team on how the proportion of PROs collected had increased. Mean presession, postsession, and change scores were presented, demonstrating the clinically meaningful impact of MT on PROs (eg, greater than 2-unit [30,31] reductions in pain, stress, and anxiety within a single MT session). Members of the MT team shared their strategies for collecting PROs among patients who were frustrated; were withdrawn or hesitant to communicate; could not speak but could communicate in other ways; were tangential in conversation; or had challenges understanding the purpose or meaning of the PRO. Additionally, the expressive therapies program manager provided individualized feedback, as needed, to members of the MT team who had lower rates of PRO collection. This individualized feedback included a discussion of practical language skills that the therapist could integrate within their routine verbal assessment and evaluation strategies used within MT sessions.

#### Study

The processes detailed above within the study section of PDSA Cycle 1 were repeated to determine rates of PRO collection following the second series of trainings. A substantial improvement in rates of PRO collection was seen across all outcomes. During the feedback sessions, members of the MT team discussed the importance of incorporating PRO assessment within their therapeutic style and asking patients to focus on the present moment when rating their stress, pain, anxiety, and coping. One area of challenge was responding to patients who asked why these PROs were being assessed. In these situations, the MT team recommended discussing the importance of the outcomes information for understanding how the patient was feeling and responding to the MT intervention.

#### Act

In reviewing the documentation from EMMPIRE Aims 1 and 2, it became clear that additional modifications to the MT EHR documentation template were needed. Therefore, a new MT EHR documentation template proposal was created that included the following modifications: (1) adding new radio button fields for conflict of service reason, session type (ie, 1-on-1 or group), intervention delivery (ie, in-person or digital), stress NRS, coping NRS, nausea NRS, and the Faces, Legs, Activity, Cry, Consolability scale for assessing pain behavior; (2) converting free-text fields (eg, session goal, MT interventions, and UTA) to checkbox fields for improved data clarity; (3) incorporating items from the inpatient psychiatry group flowsheet within the MT EHR documentation template; and (4) incorporating branching logic to enable the MT EHR documentation template to populate based on the type of session provided. This change also minimized the number of extra fields the therapist would need to complete in charting outcomes.

### Data Analysis

**Figure 2:**
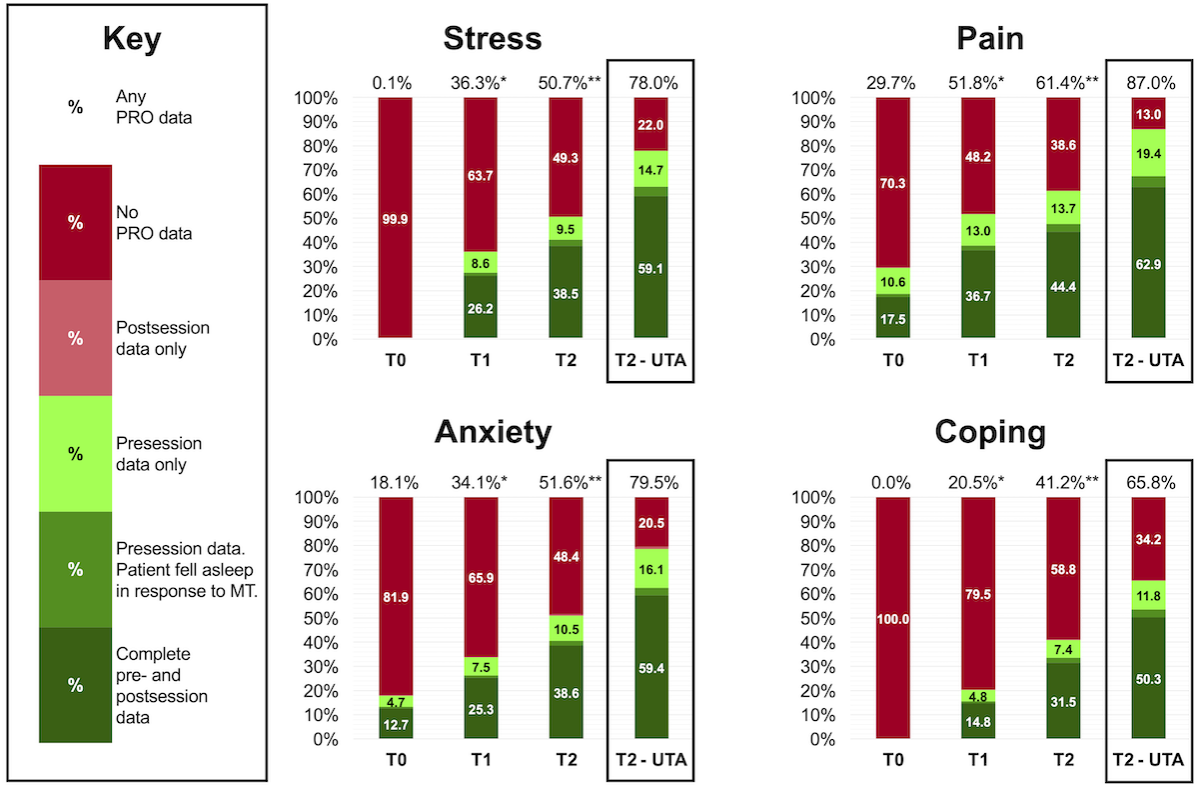
Patient-reported outcome completion rates among the music therapy team. T0 represents 2758 MT sessions documented in the 6 months before Plan-Do-Study-Act (PDSA) Cycle 1. T1 represents 2786 MT sessions documented during the 4 months of PDSA Cycle 1. T2 represents 775 MT sessions documented during the 2 months of PDSA Cycle 2. “*” indicates a statistically significant difference (*P*<.001; Fisher exact test) between rates of patient-reported outcome (PRO) collection at T0 and T1. “**” indicates a statistically significant difference (*P*<.001, Fisher exact test) between rates of PRO collection at T1 and T2. MT: music therapy; PRO: patient-reported outcomes; UTA: unable to be assessed.

The analysis compared therapists’ rates of capturing PROs during 3 discrete periods of MT documentation: (1) 2758 MT sessions documented in the 6 months before PDSA Cycle 1 (T0); (2) 2786 MT sessions documented during the 4 months of PDSA Cycle 1 (T1); and (3) 775 MT sessions documented during the 2 months of PDSA Cycle 2 (T2). For each period, patterns of collecting each PRO (ie, stress, pain, anxiety, and coping) were coded based on 6 different types of PRO completion, which are color-coded in Figure 2. Each session was coded as either having (1) complete pre- and postsession data; (2) presession data and the patient fell asleep in response to MT; (3) presession data only; (4) postsession data only; (5) no PRO data; or (6) any PRO data (either presession, postsession, or both). The completion proportion rates were calculated as the total number of sessions with the 6 PRO codes data divided by the total number of MT sessions. A Fisher exact test was used to compare therapists’ rates of collecting any PRO data and complete pre- and postsession data (1) between T0 and T1 and (2) between T1 and T2.

To account for MT sessions in which PROs were UTA, counts and percentages of reasons outcomes were UTA were calculated during T2. Adjusted completion rates were also calculated where the total number of sessions with the 6 PRO codes was divided by the total number of MT sessions that did not have outcomes UTA. Finally, to understand sessions where only presession pain data were collected at T2, counts and percentages of sessions were tallied where (1) only MT assessment and education were provided; (2) the presession pain score was 0/10 (ie, no pain intensity); and (3) the therapist noted there was a viable reason the postsession score could not be collected.

## Results

Figure 2 provides a graphical depiction of PRO completion rates among the MT team. Therapists’ rates of capturing any PRO within MT sessions increased significantly (*P*<.001) from T0 to T1 and from T1 to T2 for all domains, including stress (4/2758, 0.1% at T0; 1012/2786, 36.3% at T1; and 393/775, 50.7% at T2), pain (820/2758, 29.7% at T0; 1444/2786, 51.8% at T1; and 476/775, 61.4% at T2), anxiety (499/2758, 18.1% at T0; 950/2786, 34.1% at T1; and 400/775, 51.6% at T2), and coping (0/2758, 0% at T0; 571/2786, 20.5% at T1; and 319/775, 41.2% at T2).

Similarly, therapists’ rates of capturing complete pre- and postsession PROs within MT sessions also increased significantly (*P*<.001) from T0 to T1 and from T1 to T2 for all domains, including stress (4/2758, 0.1% at T0; 730/2786, 26.2% at T1; and 298/775, 38.5% at T2), pain (482/2758, 17.5% at T0; 1022/2786, 36.7% at T1; and 344/775, 44.4% at T2), anxiety (351/2758, 12.7% at T0; 705/2786, 25.3% at T1; and 299/775, 38.6% at T2), and coping (0/2758, 0% at T0; 411/2786, 14.8% at T1; and 244/775, 31.5% at T2).

During T2, 106/775 (13.7%) MT sessions only had presession pain data. Of these 106 sessions, 79 (74.5%) were sessions where only assessment and education were provided, 10 (9.4%) were sessions where the presession pain score was 0/10, 7 (6.6%) were sessions where the therapist noted there was a reason the postsession score could not be collected (eg, interruption or decline), and 10 (9.4%) were sessions where a reason for the missing postsession score was not documented.

During T2, the MT team reported outcomes UTA in 295/775 (38.1%) MT sessions. Reasons outcomes were UTA within MT sessions included patients’ cognitive limitations (82/295, 27.8%), PROs not applicable to the MT session (45/295, 15.3%), patients declining to provide PROs (39/295, 13.2%), patients experiencing emotional distress (36/295, 12.2%), patients’ physical limitations (33/295, 11.2%), or other reasons not specified (60/295, 20.3%). After removing MT sessions in which outcomes were UTA from T2, therapists’ rates of collecting any PRO within MT sessions were as follows: stress (393/504, 78%), pain (476/547, 87%), anxiety (400/503, 79.5%), and coping (319/485, 65.8%).

## Discussion

The purpose of this study was to determine if it was possible to improve documentation consistency and increase the capture of PROs within a medical MT team. The processes spanned every point of MT clinical care, including the training of the therapist, patient assessment and evaluation, and documentation. Through these processes, PRO collection improved throughout 10 medical centers among a team of 13.3 music therapists and MT interns. By placing all the data within the same MT EHR documentation template, the need for additional documentation burden was avoided (ie, documenting in multiple EHR sections or copying outcomes to a separate spreadsheet) as therapists implemented new skills to obtain PROs. Additionally, the documentation immediately improved without having to wait for the EHR team to build a new documentation template.

While PROs are essential elements in evaluating patients’ responses to nonpharmacologic therapies such as acupuncture, massage, and meditation [6], there are several clinical situations in which it is difficult or impossible for patients to provide PROs due to factors including physical, cognitive, or emotional limitations. Feedback from the MT team and our statistical analyses demonstrate the importance of accounting for these situations when evaluating rates of inpatient PRO collection. Additionally, it is important to understand sessions in which only the presession score is collected. Postsession scores are not appropriate in the context of assessment and education sessions where an MT intervention (eg, active music making, music-assisted relaxation, and imagery) is not delivered. Additionally, some therapists may not see the need to collect a postsession score when the patient reports presession stress, pain, or anxiety at 0/10. Since it is possible that patients could report worse scores (ie, >0) after any intervention, it is critical to ensure that therapists routinely attempt to collect postsession scores except when the patient has fallen asleep or presents with limitations as noted above.

Importantly, while rates of PRO collection continually improved over the course of the study, the rates never reached 100% of all MT sessions, even after adjusting for sessions in which outcomes were UTA. We recognize that therapists may inadvertently leave PROs out of their assessments or forget to document the reasons why outcomes were UTA. Even after accounting for UTA, the continued gap in PRO collection as demonstrated in Figure 2 (ie, stress 22%, pain 13%, anxiety 20.5%, and coping 34.2%) suggests that other factors may limit PRO collection. These factors could include (1) an education gap among new employees and interns; (2) the complex nature of individualized MT sessions among critically ill patients where specific UTA reason categories may be difficult to determine; and (3) documentation error within the EHR. Continuing education and monitoring will be needed to maintain high PRO collection rates. This continuing education will include the clinical expectation to either collect and document PROs as described in our first PDSA cycle or document why outcomes were UTA.

Our processes aligned with best practices recommended in previous studies of PRO implementation within health systems. These processes included (1) targeting multiple points in therapists’ treatment delivery and decision-making [17]; (2) integrating small systematic changes within clinical tools and resources that were already established within therapists’ routine practice [13]; (3) incorporating the MT team’s suggestions for improvement throughout the process [13]; (4) providing feedback to therapists regarding their PRO collection during monthly team meetings [11]; (5) emphasizing the value of PRO collection and the skills needed for PRO assessment and evaluation [7]; (6) building PROs within the MT EHR documentation template to facilitate data collection and provide data regarding patients’ symptoms to the medical team [6]; (7) engaged leadership [6]; (8) developing infrastructure to streamline PRO collection, data storage, extraction, and analysis [10]; (9) minimizing patient burden through brief NRS assessments [32]; and (10) continuously monitoring data completion and quality to ensure data could be stored, extracted, and used for research [8]. Additional recommendations for successful PRO collection are shown in Table 1.

**Table 1:** Recommendations for incorporating patient-reported outcomes within a medical music therapy team.

Category	Recommendation
EHR^a^ enhancement	Request enhancements to EHR documentation early, as the process for implementing changes within the EHR can take several months. Supplement the request for EHR documentation changes with a request for an on-demand report of all documentation fields within each session. If fields that are relevant to clinical practice already exist within the EHR (eg, FLACC^b^ scale, pain NRS^c^, observed emotional state, verbalized emotional state), request that these fields be added to your documentation template.
Training	When a new process for documentation is initiated, ensure therapists are provided with clear and consistent training. Monitor documentation completion and consistency on a regular basis. Implement any retraining as needed. Maintain consistent and open communication with team members to ensure questions are addressed and suggestions for improvement are implemented.
Data collection	Minimize documentation burden by capturing all data within one form. Use acronym expansions or dot phrases to create structured data entry within free-text fields if no structured data entry fields exist. When fields are not available for specific variables or outcomes, use regular expression functions within statistical packages to mine them from narrative portions of the documentation. In the pursuit of clean, discrete data on music therapy sessions, continue to provide space for therapists to write a narrative on the more qualitative and nuanced aspects of the music therapy session. Provide additional tools to facilitate therapists’ data collection such as a field note and a laminated form for patients to complete. Provide tools for therapists to document sessions in which PROs^d^are unable to be assessed due to patient limitations. Account for these sessions when calculating PRO collection rates.

^a^EHR: electronic health record.

^b^FLACC: faces, legs, activity, cry, consolability.

^c^NRS: numeric rating scale.

^d^PRO: patient-reported outcome.

Regarding limitations, our analysis of PRO collection over time is limited by a lack of data on UTA reasons at T0 and T1. It is possible that other researchers seeking to replicate our success may not have the equivalent access to EHR tools and documentation templates that were available to the MT team in our health system. This study did not consider other quality improvement approaches used in previous studies of PRO implementation, such as conducting formal interviews with music therapists to assess barriers and facilitators to PRO collection [33], recruiting clinical champions at the UH medical centers to facilitate PRO collection [7,34], developing video-based simulations modeling various techniques therapists could use to verbally collect and discuss PROs [34], or providing patients with a visual interpretation of PROs [35]. The number and frequency of meetings used to conduct this quality improvement initiative may not be feasible for other medical MT teams, and music therapists seeking to replicate these methods will need to consider their own capacity for holding these trainings. However, the trainings described in this report were conducted within the normal meeting schedule of the MT team. Given the importance of PROs for demonstrating the clinical effectiveness of MT to hospital stakeholders, temporarily increasing meeting frequency to improve PRO collection is justified.

Strengths of this study include having a baseline assessment of PRO collection, using PDSA cycle processes for quality improvement, monitoring PRO collection in real time, and minimizing documentation burden by capturing all data within the EHR. Furthermore, although the process-improvement procedures used in this study were applied to a nonpharmacologic MT intervention, the principles are applicable to numerous clinical therapists and providers (eg, nurses) in the inpatient setting.

This process-improvement study supports the feasibility of integrating standardized PRO collection within the clinical practice of nonpharmacologic therapies such as MT. Our training and documentation enhancements were effective at improving PRO data collection rates within a nonpharmacologic, medical MT team. For health care organizations, implementing quality improvement approaches such as these may yield similar increases in PRO collection with other clinical providers using pharmacologic or nonpharmacologic interventions. With the increased routine collection of PROs, health care organizations will be better able to (1) communicate the impact of their interventions to patients; (2) make decisions regarding which interventions to implement within inpatient care; and (3) demonstrate the value of nonbillable nonpharmacologic modalities such as MT.

With the Joint Commission inspiring health care systems to increase the availability of nonpharmacologic interventions (eg, acupuncture, massage therapy, meditation, and MT) for pain relief in hospitalized patients [3], routine collection of PROs will provide the opportunity to assess the effectiveness of these therapies at the individual and population level. Future research should seek to evaluate (1) whether these quality improvement approaches can be applied within medical MT teams at other health systems to improve PRO collection; (2) the clinical effectiveness of medical MT using the PROs documented in the EHR; and (3) the potential for subsequent decreased opioid use and length of stay.

## Abbreviations

EHR: electronic health record
EMMPIRE: Effectiveness of Medical Music therapy Practice: Integrative Research using the Electronic health record
FTE: full-time equivalent
MT: music therapy
NRS: numeric rating scale
PDSA: Plan-Do-Study-Act
PRO: patient-reported outcome
SPACE: stress, pain, anxiety, coping, education
UH: University Hospitals
UHCWH: University Hospitals Connor Whole Health
UTA: unable to be assessed

## Data Availability

The data sets generated and analyzed during this study are not publicly available due to privacy restrictions as the databases contain information that could compromise the privacy of research participants. However, the deidentified data sets are available from the corresponding author on reasonable request.
